# Myocardial Expression of Macrophage Migration Inhibitory Factor in Patients with Heart Failure

**DOI:** 10.3390/jcm6100095

**Published:** 2017-10-13

**Authors:** Julia Pohl, Ulrike B. Hendgen-Cotta, Pia Stock, Peter Luedike, Hideo Andreas Baba, Markus Kamler, Tienush Rassaf

**Affiliations:** 1 Department of Cardiology and Vascular Medicine, West German Heart and Vascular Center, University Hospital Essen, Hufelandstraße 55, 45147 Essen, Germany; ulrike.hendgen-cotta@uk-essen.de (U.B.H.-C.); pia.stock@uk-essen.de (P.S.); peter.luedike@uk-essen.de (P.L.); tienush.rassaf@uk-essen.de (T.R.); 2 Department of Pathology, University Hospital Essen, Hufelandstraße 55, 45147 Essen, Germany; hideo.baba@uk-essen.de; 3 Department of Thoracic Transplantation, West German Heart and Vascular Center, University Hospital Essen, Hufelandstraße 55, 45147 Essen, Germany; markus.kamler@uk-essen.de

**Keywords:** MIF, cardiomyopathy, biopsy, inflammation, oxidative stress

## Abstract

Macrophage migration inhibitory factor (MIF) is a pleiotropic inflammatory protein and contributes to several different inflammatory and ischemic/hypoxic diseases. MIF was shown to be cardioprotective in experimental myocardial ischemia/reperfusion injury and its expression is regulated by the transcription factor hypoxia-inducible factor (HIF)-1α. We here report on MIF expression in the failing human heart and assess myocardial MIF in different types of cardiomyopathy. Myocardial tissue samples from *n* = 30 patients were analyzed by quantitative Real-Time PCR. MIF and HIF-1α mRNA expression was analyzed in myocardial samples from patients with ischemic (ICM) and non-ischemic cardiomyopathy (NICM) and from patients after heart transplantation (HTX). MIF expression was elevated in myocardial samples from patients with ICM compared to NICM. Transplanted hearts showed lower MIF levels compared to hearts from patients with ICM. Expression of HIF-1α was analyzed and was shown to be significantly increased in ICM patients compared to patients with NICM. MIF and HIF-1α mRNA is expressed in the human heart. MIF and HIF-1α expression depends on the underlying type of cardiomyopathy. Patients with ICM show increased myocardial MIF and HIF-1α expression.

## 1. Introduction

Heart failure remains one of the most prevalent and challenging medical conditions with high morbidity and mortality despite advances in treatment [1]. Heart failure develops when a cardiac injury or insult impairs the ability of the heart to pump blood and maintain tissue perfusion [2,3]. A possible way to classify heart failure with reduced ejection fraction is to distinguish between ischemic (ICM) and non-ischemic cardiomyopathy (NICM) [1]. The most obvious difference between ICM and NICM is the existence of atherosclerotic lesions of the coronary arteries in patients with ICM and the absence of such lesions in patients with NICM. This leads to major differences in the initiation and the development of the cardiomyopathy and its progression to heart failure: most patients with ICM suffered at least once from myocardial infarction and they were expected to suffer from chronic ischemia due to repeated injuries occurring over time [4]. Such mechanisms do not exist in NICM. NICM consists of a heterogeneous group of etiologies including inflammatory, toxic, metabolic, genetic, valvular, hypertensive, and pericardial reasons [1]. Despite the underlying etiology, initial and ongoing myocardial injury as well as the initiation of compensatory mechanisms result in cellular damage and associated activation of an inflammatory response [5,6,7].

Macrophage migration inhibitory factor (MIF) is known as a pleiotropic inflammatory cytokine and has been recognized as a mediator of a number of inflammatory diseases including sepsis and atherosclerosis [8,9,10,11,12,13]. It has recently been described that MIF plays a role in NICM, since expression of MIF in myocardial biopsy samples predicted all-cause mortality in NICM patients and was described as a novel additional tool to predict adverse outcome in patients with NICM [14]. Importantly, MIF is not only a mediator of inflammatory processes, but also a key player in myocardial ischemia and reperfusion injury and has recently been identified as a potent cardioprotective factor [15,16,17,18,19]. Cardioprotection by MIF is a multifactorial phenomenon and is mediated by AMP-activated protein kinase (AMPK) signaling, inhibition of pro-apoptotic cascades, and attenuation of oxidative stress in the post-ischemic heart [15]. In patients with acute myocardial infarction, circulating MIF was rapidly released and MIF protein levels were found elevated [20,21].

Secretion of MIF was shown to be mediated by different stimuli like oxidative stress, ischemia, reperfusion, and hypoxia [17,19,20]. Hypoxia was not only shown to stimulate MIF protein secretion, but to influence MIF expression. MIF gene expression is subjected to induction by its transcription factor hypoxia-inducible factor (HIF)-1α [22]. HIF-1α is an oxygen-sensitive transcription factor that enables organisms to adapt to hypoxia by transcriptional activation of up to 200 genes and is considered to be the master switch of hypoxic and ischemic signaling [23]. To date, the HIF-1α/MIF axis has been investigated in multiple experimental models including cardiomyocyte and smooth muscle cell cultures after induction of hypoxia, in mouse models of myocardial ischemia/reperfusion injury, and in cancer [20,24,25,26]. All studies shared the fact that hypoxia induced MIF gene and protein expression and in some of these studies, this was due to HIF-1α-mediated signaling. Importantly, the HIF-1α/MIF axis was not the only way to stimulate hypoxia-mediated MIF signaling, since other mediators like the transcription factors NF-κB and C/EBP were discussed [26].

To date, no studies exist that report on the HIF-1α/MIF axis in heart failure. We therefore investigated MIF and HIF-1α expression in myocardial samples from patients with heart failure and show different expression levels depending on the underlying type of cardiomyopathy.

## 2. Methods

### 2.1. Study Setting and Population

Thirty patients were included in this study. Of those, 10 patients presented with end-stage ICM, 10 patients presented with end-stage NICM, and 10 patients one year after heart transplantation (HTX) due to end-stage ICM served as controls. All samples were taken from retained samples that were no longer necessary for diagnosis or clinical examinations.

Samples of *n* = 10 patients with end-stage ICM were taken directly before left ventricular assist device (LVAD) implantation. ICM patients suffered from end-stage heart failure with severe coronary artery disease (CAD) without any possibility for revascularization. Left ventricular ejection fraction (LV-EF) among this cohort was 22 ± 3% (Table 1). ICM patients presented with symptomatic heart failure (New York Heart Association (NYHA) functional class ≥II).

Myocardial biopsy samples from *n* = 10 patients with end-stage NICM (50% of NICM patients showed positive virus serology (Table 2)) were compared to those from ICM patients. CAD was excluded by coronary angiography in all of them. LV-EF was 28 ± 4% (Table 1). NICM patients presented with symptomatic heart failure (NYHA functional class ≥ II).

Myocardial samples from *n* = 10 patients after HTX served as controls. All patients underwent HTX due to end-stage ICM and myocardial biopsy was performed routinely one year after HTX. None of the HTX patients showed signs of heart failure (NYHA functional class ≤ I) and LV-EF was 56 ± 6%. None of the patients showed signs of organ rejection classified after International Society for Heart and Lung Transplantation (ISHLT) consensus report (Table 2) [27]. All patients were on a standard immunosuppressive regimen consisting of tacrolimus, MMF, and cortisone.

The study conformed to the principles outlined in the Declaration of Helsinki and was approved by local ethics committee of the University Duisburg-Essen, Germany. Key inclusion criteria were age ≥18 years. Key exclusion criteria was ongoing infectious disease with increased inflammatory parameters and/or clinical signs for infection.

### 2.2. Sample Preparation

Myocardial biopsy samples were taken from the septum of the right ventricle or from the excluded left ventricular apex directly before LVAD implantation patients.

### 2.3. RNA Isolation and Quantitative Real-Time PCR

Total RNA was extracted using RNeasy Fibrous Tissue Kit (Qiagen, Ratingen, Germany) and gene expression of MIF (primer Hs00236988_g1) and HIF-1α (primer Hs00153153_m1) was assessed by quantitative real-time PCR (qRT-PCR) using Applied Biosystems 7500 fast real-time PCR system (Applied Biosystems, Foster City, CA, USA) and normalized to glyceraldehyde-3-phosphate dehydrogenase (GAPDH) as housekeeping gene (primer Hs03929097_g1).

## 3. Statistics

Gene expression was determined by a relative quantification method. The expression of the selected genes of interest (MIF and HIF-1α) was normalized to that of GAPDH as a housekeeping gene. The statistical significance of the differences in target mRNA expression level was analyzed with the Relative Expression Software Tool (REST^®^) as described previously [28]. REST^®^ calculates the relative expression ratios on the basis of group means for the target genes versus the reference genes and tests the group ratio results for significance. In the results section, the factor of up- or downregulation of MIF and HIF-1α gene expression between the designated groups is shown.

Data are expressed as mean ± standard deviation. For clinical characteristic, the Kolmogorov–Smirnov test was applied to check for normality distribution. We used Student’s *t*-test for continuous variables. Pearson’s correlation coefficient was calculated to analyze the association between MIF and HIF-1α mRNA levels after normalization to GAPDH as housekeeping gene. Statistical analysis was performed using Prism 6.0 software (GraphPad, La Jolla, CA, USA). A *p*-value < 0.05 was considered statistically significant.

## 4. Results

### 4.1. MIF and HIF-1α Expression is Increased in Ischemic Cardiomyopathy

**Figure 1:**
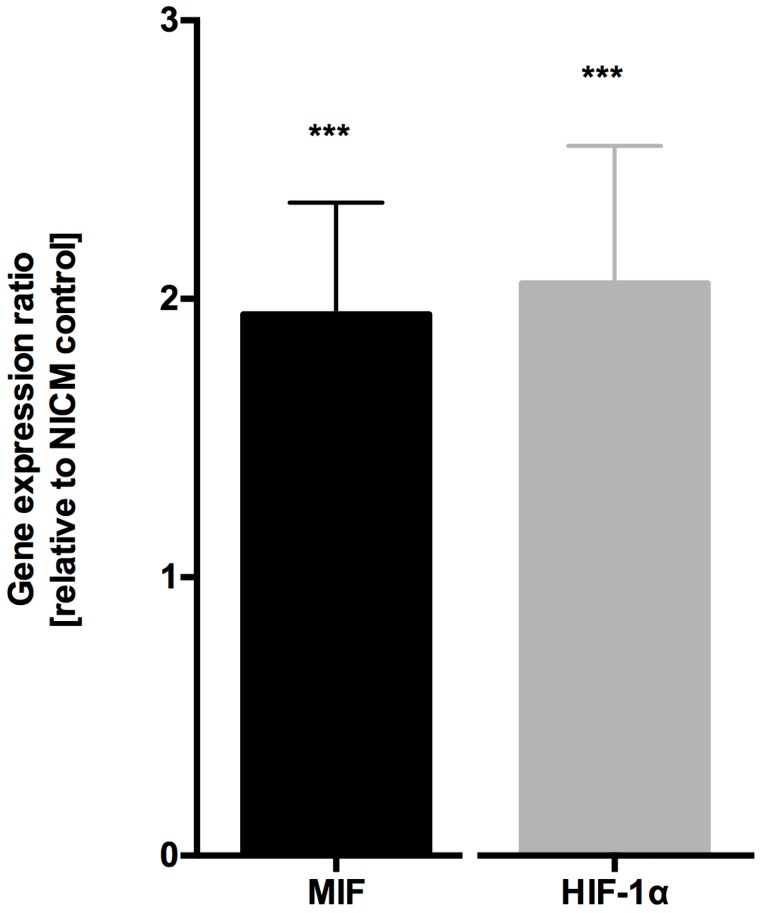
MIF and HIF-1α mRNA expression is increased in ischemic cardiomyopathy compared to non-ischemic cardiomyopathy. Myocardial MIF and HIF-1α mRNA expression were significantly increased in samples from patients with ICM compared to samples from NICM patients. Data were normalized to GAPDH and represented as relative expression ratios between samples from ICM patients and NICM patients as controls. *** *p* < 0.001 obtained with REST^®^ randomization test for MIF or HIF-1α mRNA expression in ICM samples versus samples from NICM patients.

MIF mRNA expression in myocardial samples from patients with ICM was doubled compared to samples from patients with NICM (1.944 ± 0.63, *p* < 0.001, *n* = 10, Figure 1). Since HIF-1α is the key player in acute and chronic hypoxic signaling pathways and is one of the transcription factors to regulate MIF expression [24,29], we next analyzed HIF-1α expression. In myocardial samples from ICM patients, HIF-1α expression was increased compared to myocardial samples from NICM patients (2.056 ± 0.79, *p* < 0.001, *n* = 10, Figure 1).

**Figure 2:**
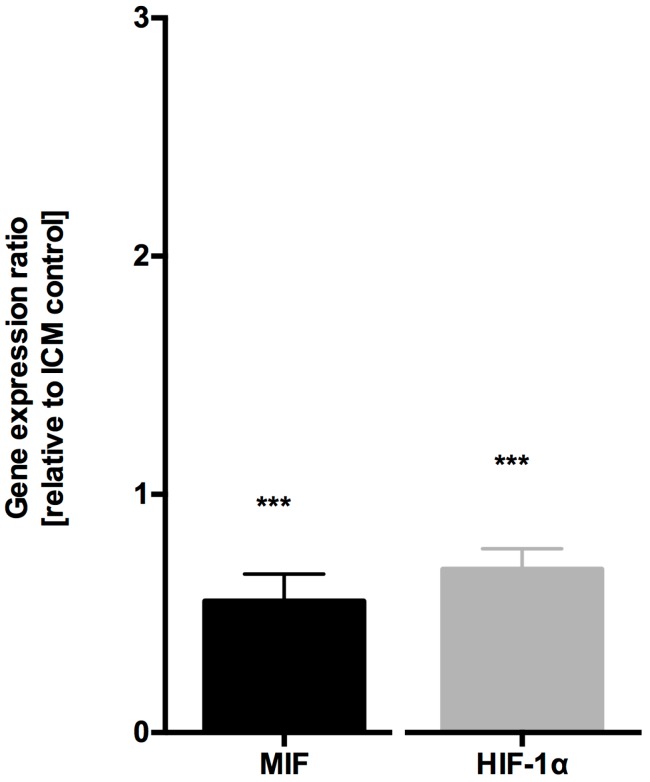
MIF and HIF-1α mRNA expression is decreased in patients after HTX compared to ischemic cardiomyopathy. Myocardial MIF and HIF-1α mRNA expression were significantly decreased in samples from patients after HTX compared to samples from ICM patients. Data were normalized to GAPDH and represented as relative expression ratios between samples from HTX patients and ICM patients as controls. *** *p* < 0.001 obtained with REST^®^ randomization test for MIF or HIF-1α mRNA expression in HTX samples versus samples from ICM patients.

Comparing MIF expression levels in myocardial samples from patients with end-stage ICM with samples from transplanted hearts with normal LV-EF, MIF mRNA expression was downregulated (0.552 ± 0.36, *p* < 0.001, *n* = 10, Figure 2). Similar results were found when comparing HIF-1α mRNA expression in samples from ICM patients with those after HTX (0.685 ± 0.25, *p* < 0.001, *n* = 10, Figure 2). There were no differences in MIF and HIF-1α expression levels when comparing samples from patients with NICM with those after HTX (data not shown).

### 4.2. MIF and HIF-1α Expression Show a Close Correlation in Myocardial Tissue Samples

**Figure 3:**
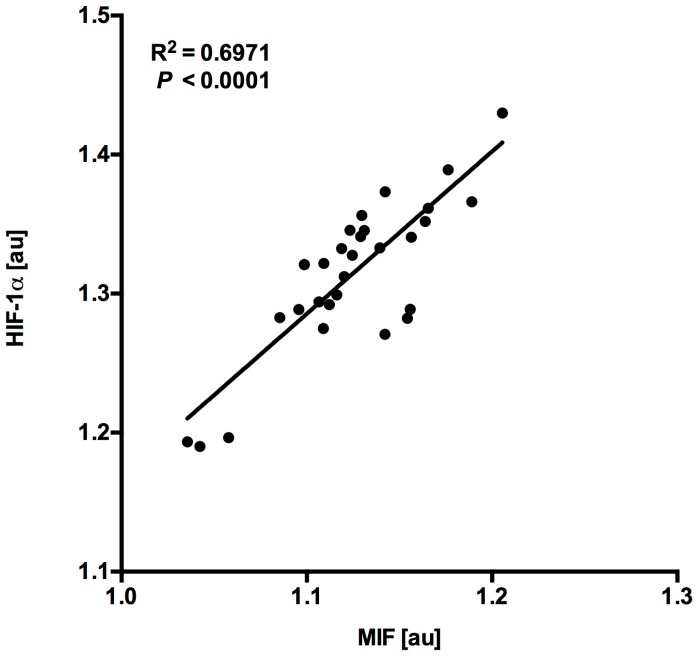
MIF expression levels correlate with HIF-1 expression levels in the human heart. MIF mRNA expression levels correlate with HIF-1 mRNA expression levels in all myocardial tissue samples (*R*^2^ = 0.6971, *p* < 0.0001, *n* = 30) after normalization to GAPDH mRNA expression levels. *au* = *arbitrary units*.

MIF and HIF-1α expression levels in myocardial samples showed a close correlation (*R*^2^ = 0.6971, *p* < 0.0001, *n* = 30, Figure 3) hinting at an association with hypoxia-induced HIF-1α expression with consecutive MIF gene regulation in cardiomyopathy.

## 5. Discussion

The present study is the first to prove MIF mRNA expression in the failing human heart and the first to describe variable expression patterns depending on the etiology of cardiomyopathy. Myocardial MIF mRNA expression is increased in end-stage ICM compared to end-stage NICM and compared to transplanted hearts with normal LV-EF pointing at an ischemia/hypoxia-mediated regulation of MIF expression in failing human hearts.

The reason why MIF is upregulated in ICM may relate to three different properties of MIF: First, MIF is a redox-regulating myocardial enzyme [18,19] and its increased expression could be interpreted as a reaction to increased oxidative stress due to chronic ischemia and reperfusion. Second, MIF is a regulator of apoptotic processes via the inhibition of the stress kinase c-Jun *N*-terminal kinase (JNK) which is activated by myocardial ischemia and reperfusion [30]. Third, MIF was described as an angiogenic factor in experimental models of myocardial ischemia and reperfusion and was attributed to mitogen-activated protein kinase (MAPK) and phosphoinositide 3-kinase (PI3K) signaling [31,32]. The ‘angiogenesis theory’ is emphasized by the parallel increase of HIF-1α expression. HIF-1α is one if the key players in hypoxic and ischemic signaling and has been shown to regulate the expression of cardiac proteins like brain and atrial natriuretic peptides [33]. Furthermore, HIF-1α acts as a transcription factor for MIF and the activation of the HIF-1α/MIF axis leads to enhanced angiogenesis [22,34]. Of course, these theories remain speculative, since our data are just delineative and the study was not designed to prove such a mechanism.

The major limitation of this trial is the lack of an appropriate internal control group, e.g., samples from patients without any signs of cardiomyopathy. Undoubtedly, direct measurement of protein expression levels in samples from healthy human hearts would be ideal, but the sampling of healthy tissue raises major ethical concerns and therefore is not feasible. In this trial, samples from transplanted hearts served as “control”. They were characterized by a normal LV-EF and histological analysis showed no signs of acute rejection. To date, there is no data on the influence of pharmaceutical immunosuppression on mRNA expression of MIF, but one study showed lowered urinary MIF protein secretion after immunosuppression in patients with glomerulonephritis [35]. Nonetheless, our main conclusion—ischemia-associated increase of myocardial MIF and HIF-1α expression—is not affected by the comparison with samples from transplanted hearts.

The second study limitation concerns our focus on gene expression. It has to be clear that this only depicts one detail in the complex biochemistry of MIF and HIF-1α signaling and the limitation to gene expression data carries the risk of missing other factors involved in the regulation of important downstream pathways. To complete the examination of the role of MIF in heart failure, it would be necessary to investigate MIF protein expression by Western blot and its localization by immunohistochemistry in myocardial samples and to amend and correlate levels of circulating MIF in plasma. Third, our data are just descriptive and do neither give a mechanistical insight into the role of the HIF-1α/MIF axis in heart failure nor provide data on outcome and prognosis. Therefore, in addition to our “proof-of-concept” data, a prospective study should be designed to investigate the aforementioned parameters and to link them to prognosis and outcome of the included patients. Future studies could then form the basis for therapeutic approaches.

Despite these study limitations, our results provide important new aspects on MIF in heart failure. Besides increased MIF expression in chronic NICM [14], our data demonstrate increased MIF expression in chronic ICM, which seems to be induced by the master switch of hypoxia HIF-1α. 

We can assume that MIF expression is not only relevant in the human heart suffering from NICM, but also in hearts from patients with ICM. Our study gives new insight into the role of MIF in heart failure and thereby complements prior studies to elucidate the “MIF story” in heart failure.

## Figures and Tables

**Table 1:** Patients’ characteristics.

Parameters	HTX	NICM	ICM	*p*-Value	*p*-Value
				(HTX vs. ICM)	(ICM vs. NICM)
*n*	10	10	10		
Sex (men)	7	8	7	1	1
Age (years)	59 ± 5	60 ±14	56 ± 8	0.3712	0.4953
NYHA class	1 ± 0	3 ± 1	4 ± 0.5	<0.0001	0.0081
LV-EF (%)	57 ± 6	28 ± 4	22 ± 6	<0.0001	0.1212
Creatinine (mg/dL)	2.3 ± 1.5	1.1 ± 0.4	1.3 ± 0.3	0.0324	0.2066
White blood cell count (×1000/µL)	6 ± 3	8 ± 3	8 ± 3	0.0938	0.7805
CRP (mg/dL)	1.4 ± 0.6	2.2 ± 1	5 ± 3.7	0.3288	0.5048
Medication (%)					
Beta blockers	60	90	100	0.0821	0.5908
Diuretics	80	100	100	0.3292	1
ACEI/ARB	80	100	70	0.8752	0.1712

NYHA = New York Heart Association functional classification; LV-EF = left ventricular ejection fraction; CRP = C-reactive protein; ACEI = angiotensin-converting-enzyme inhibitor; ARB = angiotensin receptor blocker.

**Table 2:** Histopathological criteria from myocardial biopsies.

Histopathological Criteria	HTX	NICM
Cardiac fibrosis, %	100	100
Cardiac hypertrophy, %	70	80
Positive virus serology, %	40	40
Grade of rejection, % 0R	100	na
Dallas criteria, % negative	na	100

na = not assessed; 0R = no evidence of cellular rejection.

## References

[1] Ponikowski P., Voors A.A., Anker S.D., Bueno H., Cleland J.G., Coats A.J., Falk V., González-Juanatey J.R., Harjola V.P., Jankowska E.A. (2016). 2016 ESC Guidelines for the diagnosis and treatment of acute and chronic heart failure. Eur. J. Heart Fail..

[2] Braunwald E. (2014). The war against heart failure: The Lancet lecture. Lancet.

[3] Braunwald E. (2008). Biomarkers in heart failure. N. Engl. J. Med..

[4] Kelkar A.A., Butler J., Schelbert E.B., Greene S.J., Quyyumi A.A., Bonow R.O., Cohen I., Gheorghiade M., Lipinski M.J., Sun W. (2015). Mechanisms Contributing to the Progression of Ischemic and Nonischemic Dilated Cardiomyopathy: Possible Modulating Effects of Paracrine Activities of Stem Cells. J. Am. Coll. Cardiol..

[5] Shioi T., Matsumori A., Kihara Y., Inoko M., Ono K., Iwanaga Y., Yamada T., Iwasaki A., Matsushima K., Sasayama S. (1997). Increased expression of interleukin-1 beta and monocyte chemotactic and activating factor/monocyte chemoattractant protein-1 in the hypertrophied and failing heart with pressure overload. Circ. Res..

[6] Singal P.K., Khaper N., Palace V., Kumar D. (1998). The role of oxidative stress in the genesis of heart disease. Cardiovasc. Res..

[7] Chen D., Assad-Kottner C., Orrego C., Torre-Amione G. (2008). Cytokines and acute heart failure. Crit. Care Med..

[8] Asare Y., Schmitt M., Bernhagen J. (2013). The vascular biology of macrophage migration inhibitory factor (MIF). Expression and effects in inflammation, atherogenesis and angiogenesis. Thromb. Haemost..

[9] Pohl J., Papathanasiou M., Heisler M., Stock P., Kelm M., Hendgen-Cotta U.B., Rassaf T., Luedike P. (2016). Renal replacement therapy neutralizes elevated MIF levels in septic shock. J. Intensive Care.

[10] Pohl J., Rammos C., Totzeck M., Stock P., Kelm M., Rassaf T., Luedike P. (2016). MIF reflects tissue damage rather than inflammation in post-cardiac arrest syndrome in a real life cohort. Resuscitation.

[11] Luedike P., Rammos C., Pohl J., Heisler M., Totzeck M., Kleophas W., Hetzel G.R., Kelm M., Hendgen-Cotta U., Rassaf T. (2015). Filtration of Macrophage Migration Inhibitory Factor (MIF) in Patients with End Stage Renal Disease Undergoing Hemodialysis. PLoS ONE.

[12] Rammos C., Hendgen-Cotta U.B., Pohl J., Totzeck M., Luedike P., Schulze V.T., Kelm M., Rassaf T. (2014). Modulation of circulating macrophage migration inhibitory factor in the elderly. Biomed. Res. Int..

[13] Burger-Kentischer A., Goebel H., Seiler R., Fraedrich G., Schaefer H.E., Dimmeler S., Kleemann R., Bernhagen J., Ihling C. (2002). Expression of macrophage migration inhibitory factor in different stages of human atherosclerosis. Circulation.

[14] Mueller K.A., Schwille J., Vollmer S., Ehinger E., Kandolf R., Klingel K., Kramer U., Gawaz M., Geisler T., Mueller I.I. (2016). Prognostic impact of macrophage migration inhibitory factor in patients with non-ischemic heart failure undergoing endomyocardial biopsy. Int. J. Cardiol..

[15] Rassaf T., Weber C., Bernhagen J. (2014). Macrophage migration inhibitory factor in myocardial ischaemia/reperfusion injury. Cardiovasc. Res..

[16] Luedike P., Hendgen-Cotta U.B., Sobierajski J., Totzeck M., Reeh M., Dewor M., Lue H., Krisp C., Wolters D., Kelm M. (2012). Cardioprotection through S-nitros(yl)ation of macrophage migration inhibitory factor. Circulation.

[17] Miller E.J., Li J., Leng L., McDonald C., Atsumi T., Bucala R., Young L.H. (2008). Macrophage migration inhibitory factor stimulates AMP-activated protein kinase in the ischaemic heart. Nature.

[18] Koga K., Kenessey A., Powell S.R., Sison C.P., Miller E.J., Ojamaa K. (2011). Macrophage migration inhibitory factor provides cardioprotection during ischemia/reperfusion by reducing oxidative stress. Antioxid. Redox. Signal..

[19] Pohl J., Hendgen-Cotta U.B., Rammos C., Luedike P., Mull E., Stoppe C., Jülicher K., Lue H., Merx M.W., Kelm M. (2016). Targeted intracellular accumulation of macrophage migration inhibitory factor in the reperfused heart mediates cardioprotection. Thromb. Haemost..

[20] Takahashi M., Nishihira J., Katsuki T., Kobayashi E., Ikeda U., Shimada K. (2002). Elevation of plasma levels of macrophage migration inhibitory factor in patients with acute myocardial infarction. Am. J. Cardiol..

[21] Yu C.M., Lau C.P., Lai K.W.H., Huang X.R., Chen W.H., Lan H.Y. (2001). Elevation of plasma level of macrophage migration inhibitory factor in patients with acute myocardial infarction. Am. J. Cardiol..

[22] Simons D., Grieb G., Hristov M., Pallua N., Weber C., Bernhagen J., Steffens G. (2011). Hypoxia-induced endothelial secretion of macrophage migration inhibitory factor and role in endothelial progenitor cell recruitment. J. Cell Mol. Med..

[23] Heinl-Green A., Radke P.W., Munkonge F.M., Frass O., Zhu J., Vincent K., Geddes D.M., Alton E.W. (2005). The efficacy of a ‘master switch gene’ HIF-1alpha in a porcine model of chronic myocardial ischaemia. Eur. Heart J..

[24] Fu H., Luo F., Yang L., Wu W., Liu X. (2010). Hypoxia stimulates the expression of macrophage migration inhibitory factor in human vascular smooth muscle cells via HIF-1alpha dependent pathway. BMC Cell Biol..

[25] Ma H., Wang J., Thomas D.P., Tong C., Leng L., Wang W., Merk M., Zierow S., Bernhagen J., Ren J. (2010). Impaired macrophage migration inhibitory factor-AMP-activated protein kinase activation and ischemic recovery in the senescent heart. Circulation.

[26] Larsen M., Tazzyman S., Lund E.L., Junker N., Lewis C.E., Kristjansen P.E.G., Murdoch C. (2008). Hypoxia-induced secretion of macrophage migration-inhibitory factor from MCF-7 breast cancer cells is regulated in a hypoxia-inducible factor-independent manner. Cancer Lett..

[27] Stewart S., Winters G.L., Fishbein M.C., Tazelaar H.D., Kobashigawa J., Abrams J., Andersen C.B., Angelini A., Berry G.J., Burke M.M. (2005). Revision of the 1990 working formulation for the standardization of nomenclature in the diagnosis of heart rejection. J. Heart Lung Transplant..

[28] Pfaffl M.W., Horgan G.W., Dempfle L. (2002). Relative expression software tool (REST) for group-wise comparison and statistical analysis of relative expression results in real-time PCR. Nucleic Acids Res..

[29] Belaiba R.S., Bonello S., Zähringer C., Schmidt S., Hess J., Kietzmann T., Görlach A. (2007). Hypoxia up-regulates hypoxia-inducible factor-1alpha transcription by involving phosphatidylinositol 3-kinase and nuclear factor kappaB in pulmonary artery smooth muscle cells. Mol. Biol. Cell.

[30] Qi D., Hu X., Wu X., Merk M., Leng L., Bucala R., Young L.H. (2009). Cardiac macrophage migration inhibitory factor inhibits JNK pathway activation and injury during ischemia/reperfusion. J. Clin. Investig..

[31] Amin M.A., Volpert O.V., Woods J.M., Kumar P., Harlow L.A., Koch A.E. (2003). Migration inhibitory factor mediates angiogenesis via mitogen-activated protein kinase and phosphatidylinositol kinase. Circ. Res..

[32] Kupatt C., Horstkotte J., Vlastos G.A., Pfosser A., Lebherz C., Semisch M., Thalgott M., Büttner K., Browarzyk C., Mages J. (2005). Embryonic endothelial progenitor cells expressing a broad range of proangiogenic and remodeling factors enhance vascularization and tissue recovery in acute and chronic ischemia. FASEB J..

[33] Casals G., Ros J., Sionis A., Davidson M.M., Morales-Ruiz M., Jiménez W. (2009). Hypoxia induces B-type natriuretic peptide release in cell lines derived from human cardiomyocytes. Am. J. Physiol. Heart Circ. Physiol..

[34] Chesney J.A., Mitchell R.A. (2015). 25 Years On: A Retrospective on Migration Inhibitory Factor in Tumor Angiogenesis. Mol. Med..

[35] Zwiech R. (2015). Macrophage migration inhibitory factor urinary excretion revisited—MIF a potent predictor of the immunosuppressive treatment outcomes in patients with proliferative primary glomerulonephritis. BMC Immunol..

